# Severe acquired hemophilia A associated with COVID-19 vaccination: A case report and literature review

**DOI:** 10.1097/MD.0000000000039166

**Published:** 2024-08-02

**Authors:** Hong Jun Kim, Ye Ji Jung, Jun Ho Lee, Hyun Jung Lee, Chi Hoon Maeng, Sun Kyung Baek, Jae Joon Han

**Affiliations:** a Department of Hematology and Medical Oncology, Kyung Hee University, College of Medicine, Seoul, Republic of Korea.

**Keywords:** acquired hemophilia A, coronavirus disease-19, factor VIII, inhibitor, vaccination

## Abstract

**Rationale:**

Acquired hemophilia A (AHA) is a rare autoimmune disease caused by an antibody that inhibits coagulation factor VIII activity. More than half of patients with AHA cannot identify underlying disorders. The remaining patients are associated with malignancies, autoimmune diseases, skin diseases, infections, and medications. Here, we present a case of 56-year-old Korean man with underlying hypertension, dyslipidemia, and diabetes mellitus who developed AHA following the second dose of BNT162b2 COVID-19 vaccination.

**Patient concerns:**

He presented with a large 20 × 30 cm-sized hematoma along the psoas muscle and intracranial hemorrhage, necessitating intensive care with mechanical ventilation and continuous renal replacement therapy. Laboratory testing demonstrated that activated partial thromboplastin time and prothrombin times were 74.7 seconds (normal range 29–43 seconds) and 17.2 seconds (normal range 12.5–14.7 seconds), respectively.

**Diagnoses:**

Laboratory tests confirmed AHA with undetectable factor VIII activity (<1.5%) and a positive factor VIII antibody with a titer of 8.49 Bethesda units/mL.

**Interventions:**

Recombinant factor VIIa (NovoSeven^®^) was administered every 2 hours to control the bleeding, alongside immunosuppression with methylprednisolone 1 mg/kg daily and cyclophosphamide 2 mg/kg daily to eliminate the autoantibody.

**Outcomes:**

Despite the treatments, the patient developed sepsis and succumbed 14 weeks after admission.

**Lessons:**

This rare case underscores the importance of monitoring for AHA following COVID-19 vaccination. Although the benefits outweigh the risks of vaccination, AHA should be considered in the differential diagnosis of unusual bleeding following the vaccinations. Early diagnosis and management before severe bleeding are critical for successfully controlling life-threatening bleeding.

## 1. Introduction

Acquired hemophilia A (AHA) is a rare bleeding disorder caused by inhibitory autoantibodies against coagulation factor VIII (FVIII). The incidence of AHA is 1.48 cases per 1 million individuals per year, with frequency increased in pregnancy and the elderly, based on a 2-year surveillance study in the United Kingdom.^[1]^ Patients with AHA commonly experience bleeding in the skin or deep muscles, whereas those with congenital hemophilia A more frequently have joint bleeding. More than half of the patients with AHA cannot identify underlying disorders (idiopathic), and the remaining are associated with malignancies, autoimmune diseases, skin diseases, infections, and medications.^[2]^ There are only a few cases of AHA following influenza vaccinations.^[3,4]^ Recently, a few case series reported AHA after vector-based (ChAdOx1 nCoV-19) or mRNA-based (BNT162b2 and mRNA-1273) COVID-19 vaccines. Here, we report a case of AHA following the second dose of COVID-19 vaccination. The patient has provided written informed consent for publication of the case and any accompanying images.

## 2. Case description

**Figure 1.**
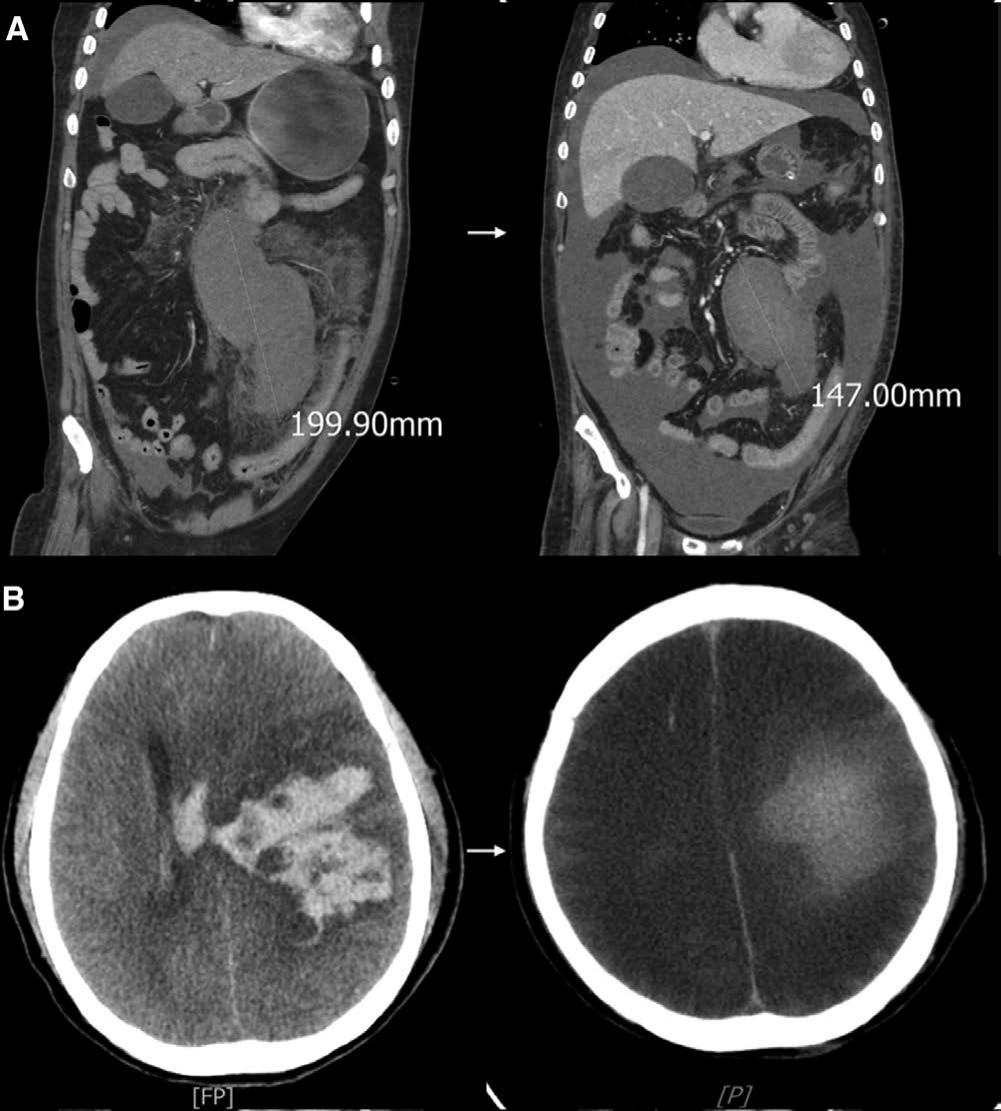
Bleeding before and after the treatment of acquired hemophilia. (A) A coronal section of the abdomen computed tomography showed the intraperitoneal hematoma spreading along the left psoas muscle (left). The size of the intraperitoneal hematoma decreased after treatment (right). (B) The transverse section of the brain computed tomography showed intracranial hemorrhage at the left basal ganglia and frontoparietal lobe with midline shifting to the right (left). The intracranial hemorrhage was improved after treatment (right).

A 56-year-old man with a history of hypertension, dyslipidemia, and diabetes mellitus was transferred to our hospital after receiving his second dose of the COVID-19 vaccine (BNT162b2) 2 weeks earlier. Upon arrival at the emergency department, he exhibited severe headaches and a drowsy mentality, along with significant abdominal distension. A notable mass was palpable in the left abdominal quadrant upon examination. His vital signs revealed a blood pressure of 95/56 mm Hg, an increased respiratory rate, and decreased urine output. Abdominopelvic computed tomography (CT) scans revealed a large 20 cm-sized hematoma along the left psoas muscle and additional intramuscular bleeding in the right iliopsoas muscle (Fig. 1A). The scans also showed extensive bleeding in the peritoneal cavity, particularly around the liver, spleen, and flanks. Laboratory tests indicated an activated partial thromboplastin time (aPTT) of 74.7 seconds and a prothrombin time (PT) of 17.2 seconds. A 1:1 mixing study revealed no significant correction of the aPTT. Initial FVIII activity was undetectable (<1.5%) and FVIII antibody test was positive, confirming the diagnosis of AHA. We considered the mild prolongation of PT results from severe coagulation factor consumption after a large amount of intraabdominal bleeding. His hemoglobin concentration was 6.1 g/dL, necessitating a transfusion of packed red blood cells. Additional significant findings included a white blood cell count of 18,300/mm³ and a creatinine level of 1.79 mg/dL (estimated glomerular filtration rate of 41.4 mL/min). His total bilirubin was 5.59 mg/dL, alanine and asparate aminotransferase was 92 and 76 U/L, respectively. A rapid decline in mental status was observed, attributed to intracranial hemorrhage in the left basal ganglia and frontoparietal lobe as detected by brain CT (Fig. 1B). After admission, an aPTT was >180 seconds and a PT was 16.9 seconds. FVIII antibody titer was 8.49 Bethesda units/mL, Mechanical ventilation and continuous renal replacement therapy were initiated in the ICU. Following transfer to our center, recombinant factor VIIa (rFVIIa; NovoSeven) was administered at 90 µg/kg intravenously every 2 hours to control active bleeding. However, persistent oral bleeding and hemoptysis through the endotracheal tube continued for several days. The treatment was switched from rFVIIa to factor 8 inhibitor bypassing activity (FEIBA, 5000 IU every 8 hours). Approximately ten weeks later, the intracranial hemorrhage had resolved, and the size of the intraperitoneal hematoma had decreased, as confirmed by follow-up brain and abdominal CT (Fig. 1).

**Figure 2.**
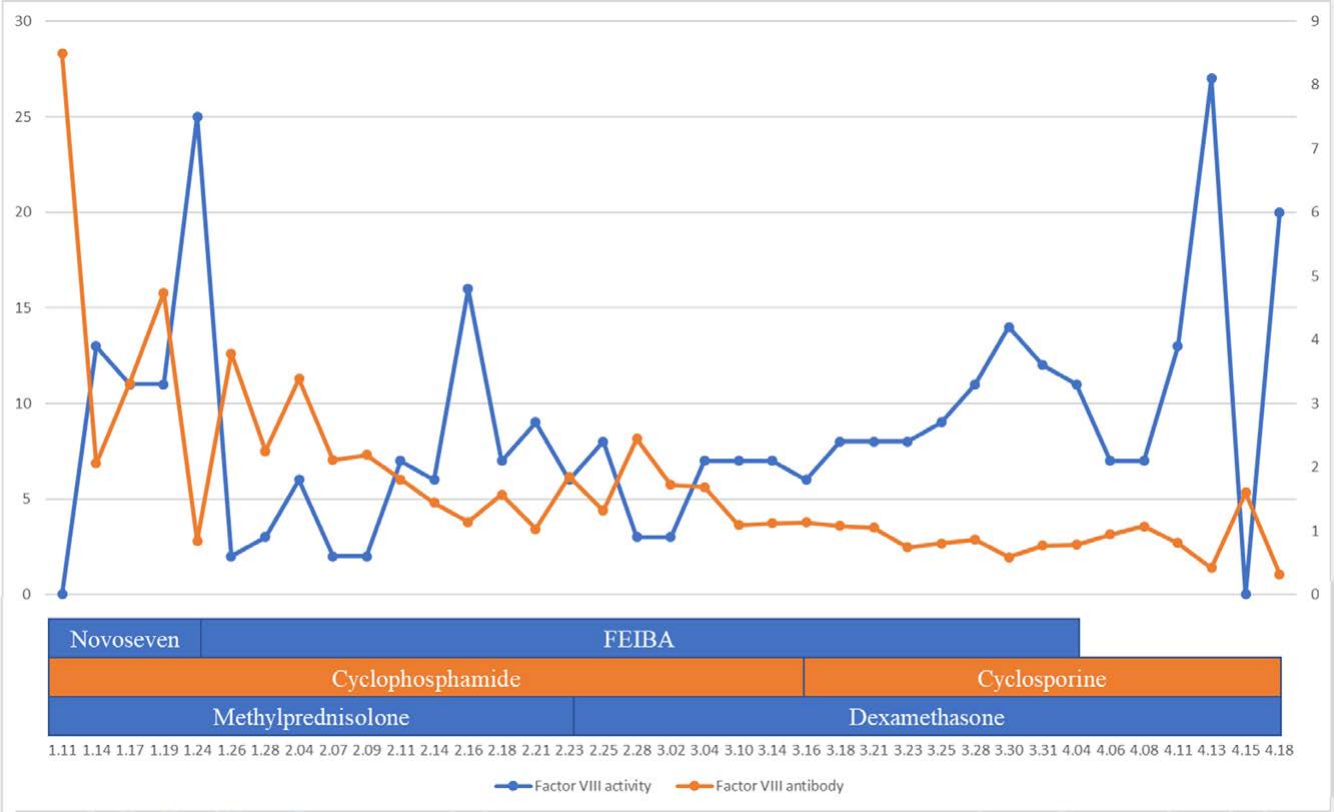
Changes over time in treatments, factor VIII activity, and factor VIII antibody levels.

Methylprednisolone at 65 mg (equivalent to prednisolone 1 mg/kg) daily and cyclophosphamide at 2 mg/kg daily were administered intravenously to eliminate the autoantibody. The titer of the FVIII inhibitor decreased to 0.84 Bethesda Units(BU)/mL, and FVIII activity increased to 25% after 2 weeks of immunosuppression. However, the titer of FVIII inhibitor rose to 1.85 BU/mL after 43 days of methylprednisolone and cyclophosphamide. We first changed methylprednisolone to dexamethasone 20 mg (approximate to prednisolone 2 mg/kg), and the dose of dexamethasone was tapered until 6mg. The titer of the FVIII inhibitor decreased to 1.13 BU/mL after 3 weeks of treatment with dexamethasone and cyclophosphamide. We decided to change cyclophosphamide to cyclosporine to eradicate FVIII inhibitors because few cases showed complete remission with cyclosporine after cyclophosphamide failure.^[5]^ After 1 month, the titer of FVIII inhibitor decreased further to 0.32 BU/mL (Fig. 2).

Despite the cessation of active bleeding, the patient succumbed to sepsis after 14 weeks of hospitalization. Cyclosporine 100 mg bid and dexamethasone 6mg was administered at the time of death. Meropenem 1 g per 8 hours was continuously administered from the admission and vancomycin 1 g per 24 hours was administered for 30 days to control the infections. However, Enterobacter cloacae complex was identified from the blood culture 5 days before the death.

## 3. Discussion

To our knowledge, this is the first reported case of AHA following COVID-19 vaccination in Korea. The patient developed AHA approximately 15 days after receiving the second dose of the BNT162b2 COVID-19 vaccine. AHA is a rare autoimmune disorder characterized by the formation of autoantibodies against coagulation FVIII, leading to impaired blood clotting and a propensity for spontaneous bleeding. The incidence of AHA is estimated at 1.48 cases per million individuals per year, with various etiologies, more than half of which are idiopathic.^[1]^ The severity of AHA can range from mild to life-threatening, depending on the extent of bleeding and associated complications. Treatment strategies for AHA typically involve the intravenous administration of bypassing agents such as rFVIIa or activated prothrombin complex concentrate to supplement clotting function, alongside immunosuppression to eradicate autoantibody production, and supportive care to manage bleeding episodes.^[2,6]^ Our patient was treated with rFVIIa followed by FEIBA to control bleeding and corticosteroids combined with cyclophosphamide to eradicate the autoantibody. However, the patient died from sepsis after a severe life-threatening bleeding episode. The clinical course of this patient highlights the importance of timely diagnosis of AHA in the context of postvaccination events. Early diagnosis and prompt initiation of treatment can significantly improve clinical outcomes. Given the rarity of AHA and its temporal relationship with vaccination, it is essential to consider the possibility of a causal link and continue monitoring for such adverse events. It is crucial to emphasize the overall safety profile of COVID-19 vaccines, which have proven highly effective in preventing severe disease, hospitalization, and death from COVID-19. The benefits of vaccination far outweigh the potential risks of rare adverse events, such as AHA. The overall incidence of AHA after vaccination remains extremely low.^[7]^ Therefore, the potential connection warrants further investigation to better understand the possible risks and underlying mechanisms. In addition, healthcare providers should remain vigilant for any unusual clinical presentations following vaccination, including prolonged aPTT and spontaneous bleeding suggestive of AHA.

When evaluating cases suggestive of AHA, it is crucial to consider other possible etiologies and risk factors. Approximately 50% of AHA cases are associated with underlying conditions, including autoimmune diseases, malignant diseases, and pregnancy.^[8,9]^ Drug-related AHA is rare, accounting for <5% of cases.^[8]^ In a study of 96 AHA cases, 21% of patients were younger than 65 years old. The presence of a preexisting disease that could explain the occurrence of AHA was documented in only 20 cases (21%).^[8,9]^ Therefore, it is essential to consider the age, bleeding characteristics, and concurrent conditions while investigating a possible connection between COVID-19 vaccination and AHA.

We identified 16 cases from a literature search of MEDLINE through PUBMED from January 2020 to December 2022 (Table 1).^[10]^ We compared the clinical characteristics of 17 AHA cases (including this case) after COVID-19 vaccination with 55 Korean AHA patients with other underlying conditions. Most patients in the COVID-19 vaccination group were older than those in the comparison group, with 88% older than 65. More patients in the COVID-19 vaccination group exhibited minor severity bleeding symptoms and lower FVIII inhibitor titers than the comparison group. The gender ratio and other clinical characteristics were similar between the 2 groups (Table 2).

**Table 1:** Seventeen cases of acquired hemophilia A following COVID-19 vaccination.

Reference number	Age	Sex	Medical history	Type of V=vaccine	No	Onset days*	Hb (g/dL)	FVIII activity	FVIII inhibitor	Bypassing agents	Immunosuppressants	Results
^[10]^	75	M	HTN, Dyslipidemia, CAD	BNT162b2	2	90	7.4	<1%	318	rFVIIa	Rituximab, CTX, Cyclosporine	After a week VIII activity increased to 20%, aPTT of 33.6 s
^[11]^	95	F	HTN, CHF, Breast ca (CR)	BNT162b2	1	7	11.1	Undetectable	5.4	rFactor VIII	PDL, Rituximab	After third dose of rituximab, FVIII inhibitor decreased to 0.7 BU/ml, normalization of FVIII level
^[12]^	80	M	DM, HTN, CKD, Dyslipidemia, CVA	BNT162b2	1	14	7.3	6.70%	7.5	rFVIIa	MPD, azathioprine, PDL	After 6 weeks of treatment, normalization of FVIII titer, FVIII inhibitors were undetectable
^[13]^	67	M	HTN, pulmonary sarcoidosis	BNT162b2	2	19	10.2	<1%	110	aPCC, rFVIIa	PDL, rituximab	After 34 days of admission normalization of FVIIIa titer, FVIIIa inhibitors were undetectable
^[14]^	86	M	Polymyalgia rheumatica	BNT162b2	2	14	6.6	FVIII:C 0.06 IU/mL	2.1		MPD	FVIII:C within the reference range, undetectable inhibitor
^[14]^	73	F	RA, Sjogren syndrome	BNT162b2	2	26	9.7	FVIII:C 0.05 IU/mL	0.8		MPD	FVIII:C within the reference range, undetectable inhibitor
^[14]^	67	M	–	BNT162b2	2	49	12.5	FVIII:C 0.06 IU/mL	2.5	rFVIIa	PDL, CTX	FVIII:C within the reference range, undetectable inhibitor
^[14]^	77	M	Bladder cancer	BNT162b2	2	52	6.6	FVIII:C 0.02 IU/mL	6.9	rFVIIa	Rituximab, MPD	FVIII:C within the reference range, undetectable inhibitor. Expired d/t severe sepsis
^[15]^	72	M	–	mRNA-1273	2	9	7.8	Undetectable	158.6	rFVIIa	PDL, CTX, rituximab	FVIII inhibitors decreased to 20.6 BU/mL, FVIII activity increased to 8%
^[16]^	70	M	Polymyalgia rheumatica, HCV antibody	mRNA-1273	1	8	9.5	0.03 IU/mL	39.9	aPCC, rFVIIa,	PDL, CTX	FVIII inhibitors decreased to 11.4 BU/mL, FVIII activity increased 0.07 IU/mL
^[17]^	77	M	–	mRNA-1273	2	21	9.4	0.60%	71.6	aPCC rFVIIa,	PDL, CTX	FVIII activity increased to 9%, FVIII inhibitor decreased to 49 BU/mL
^[18]^	85	M	HTN, CABG, PAD	mRNA-1273	1	7	–	Undetectable	2.2	rFVIIa, aPCC	PDL, rituximab	–
^[18]^	86	F	AV stenosis, 3˚ AV block s/p pacemaker	mRNA-1273	2	21	–	23%	1.01	rFVIIa, APCC	PDL	After 17days of treatment, FVIII:C increased to 178%
^[18]^	72	F	arterial disease	mRNA-1273	1	14	5.6	Undetectable	12.4	rFVIIa	PDL, rituximab	FVII activity increased to 5%, FVIII inhibitor decreased to 5.6BU/mL
^[19]^	69	M	DM, HTN, Prostate ca (remission)	BNT162b2	1	9	11.6	1%	80		PDL	After 4 weeks of treatment, FVIII level increased to 5%, FVIII inhibitor decreased to 2BU/mL
^[20]^	39	W	–	BNT162b2	1	10	8.6	2%	17.2		PDL, rituximab	after 2 months, normalization of FVIII level
Case	56	M	HTN, Dyslipidemia, DM	BNT162b2	2	15	6.1	<1.5%	8.49	rFVIIa, aPCC	MPD, CTX	ICH, Expired d/t severe sepsis

aPCC = activated prothrombin complex concentrate, CABG = coronary artery bypass grafting, CAD = coronary artery disease, CHF = congestive heart failure, CKD = chronic kidney disease, CTX = cyclophosphamide, CVA = cerebrovascular accident, DM = diabetes mellitus, HCV = hepatitis C virus, HTN = hypertension, ICH = intracerebral hemorrhage, MPD = methylprednisolone, PAD = peripheral artery disease, PDL = prednisolone, RA = rheumatoid arthritis, rFVIIa = recombinant factor VIIa.

**Table 2:** Clinical characteristics acquired hemophilia A following COVID-19 vaccination compared to 55 acquired hemophilia A in Korea from other etiologies.

Characteristics	AHA in Korea^[5]^ n = 55 (%)	AHA after COVID-19 vaccination^[10–20]^ n = 17 (%)
Age (yrs)	65 (15–86)	73 (39–95)
Age > 60 yrs	35 (63.6)	15 (88.2)
Gender (Male/Female)	36 (65.5)/19 (34.5)	12 (70.6)/5 (29.4)
Days from vaccination (days)	Not applicable	14 (7–90)
Concomitant disorders		
Coronary artery disease	0	2 (11.8)
Renal failure	1 (1.8)	1 (5.9)
Hypertension	13 (23.6)	7 (41.2)
Type 2 diabetes mellitus	12 (21.8)	3 (17.6)
Relapsed malignancy	0	1 (5.9)
Cured malignancy	3 (5.4)	2 (11.8)
Laboratory findings		
Hemoglobin (g/dL)	8.40 (3.6–16.3)	8.6 (5.6–12.5)
FVIII level (IU/dL)	1.35 (0–21.9)	Not available
FVIII inhibitor titer (BU/mL)	14.65 (0.6–175.5)	8.49 (0.8–318)
Presence of bleeding symptoms	50 (90.9)	17 (100)
Severity of bleeding		
Major	37 (74.0)	8 (47.1)
Minor	13 (26.0)	9 (52.9)
Number of bleeding sites		
1	14 (28.0)	4 (23.5)
≥2	36 (72.0)	13 (76.5)
Cause of bleeding		
Spontaneous	38 (76.0)	15 (88.2)
Trauma	7 (14.0)	2 (11.8)
Surgery	4 (8.0)	0
Peripartum	1 (2.0)	0
Bleeding sites		
Subcutaneous and skin	25 (50.0)	13 (76.5)
Muscle	19 (38.0)	3 (17.6)
Gastrointestinal tract	10 (20.0)	1 (5.9)
Mucosa	10 (20.0)	1 (5.9)
Retroperitoneum	8 (16.0)	2 (11.8)
Genitourinary tract	8 (16.0)	2 (11.8)
Joint	7 (14.0)	1 (5.9)
Intracranial	0	1 (5.9)

AHA = acquired hemophilia A.

The pathophysiology of AHA in the context of COVID-19 vaccination remains to be elucidated; however, it is thought that the immune response triggered by the vaccine might play a role in the development of autoantibodies against FVIII.^[21]^ The median of 14 days (1 week to 3 weeks in 13 patients, Table 1) from vaccination to diagnosis supports the immune reaction as the underlying mechanism. Few patients diagnosed 26, 49, 52, and 90 days after vaccination suggest that these cases were not promptly diagnosed (Table 1). Both molecular mimicry and activation of dormant lymphocytes specific to factor VIII can lead to the production of autoantibodies.^[22]^ In our analysis of 17 case series, we did not find a significant difference between the first and second vaccination or between the 2 types of vaccine products.

## 4. Conclusion

This case further highlights the potential safety concerns related to COVID-19 vaccinations. While the benefits of vaccination considerably outweigh the risks, monitoring is essential for identifying rare adverse reactions. Medical professionals must consider AHA when confronted with prolonged aPTT, with or without spontaneous bleeding, as this condition can become life-threatening without timely intervention.

## Author contributions

**Conceptualization:** Hong Jun Kim, Jae Joon Han.

**Writing – original draft:** Hong Jun Kim, Jae Joon Han.

**Visualization:** Ye Ji Jung.

**Data curation:** Jun Ho Lee.

**Software:** Hyun Jung Lee.

**Supervision:** Chi Hoon Maeng, Jae Joon Han.

**Validation:** Sun Kyung Baek, Jae Joon Han.

## References

[1] Collins PW Hirsch S Baglin TP.; UK Haemophilia Centre Doctors' Organisation. Acquired hemophilia A in the United Kingdom: a 2-year national surveillance study by the United Kingdom Haemophilia Centre Doctors’ Organisation. Blood. 2007;109:1870–7. 17047148 10.1182/blood-2006-06-029850

[2] Knoebl P Marco P Baudo F.; EACH2 Registry Contributors. Demographic and clinical data in acquired hemophilia A: results from the European Acquired Haemophilia Registry (EACH2). J Thromb Haemost. 2012;10:622–31. 22321904 10.1111/j.1538-7836.2012.04654.x

[3] Pirrotta M Bernardeschi P Fiorentini G. A case of acquired haemophilia following H1N1 vaccination. Haemophilia. 2011;5:815. 10.1111/j.1365-2516.2011.02493.x 21447116

[4] Moulis G Pugnet G Bagheri H. Acquired factor VIII haemophilia following influenza vaccination. Eur J Clin Pharmacol. 2010;66:1069–70. 20582585 10.1007/s00228-010-0852-z

[5] Hyun SY Shin HJ Yoon SS. Clinical characteristics and prognostic factors of acquired haemophilia A in Korea. Haemophilia. 2021;27:e609–16. 34156738 10.1111/hae.14370

[6] Pishko AM Doshi BS. Acquired hemophilia A: current guidance and experience from clinical practice. J Blood Med. 2022;13:255–65. 35592586 10.2147/JBM.S284804 PMC9112043

[7] Franchini M Cappello E Valdiserra G. Investigating a signal of acquired hemophilia associated with COVID-19 vaccination: a systematic case review. Semin Thromb Hemost. 2022;49:15–26. 36055265 10.1055/s-0042-1754389

[8] Coppola A Franchini M Tripodi A.; ad hoc Working Group (Appendix 1). Acquired haemophilia A: Italian consensus recommendations on diagnosis, general management and treatment of bleeding. Blood Transfus. 2022;20:245–62. 35175184 10.2450/2022.0238-21 PMC9068356

[9] Tengborn L Baudo F Huth-Kühne A.; EACH2 registry contributors. Pregnancy-associated acquired haemophilia A: results from the European Acquired Haemophilia (EACH2) registry. BJOG. 2012;119:1529–37. 22901076 10.1111/j.1471-0528.2012.03469.x

[10] Al Hennawi H Al Masri MK Bakir M. Acquired hemophilia A post-COVID-19 vaccination: a case report and review. Cureus. 2022;14:e21909. 35265430 10.7759/cureus.21909 PMC8898568

[11] Murali A Wong P Gilbar PJ. Acquired Hemophilia A following Pfizer-BioNTech SARS CoV-2 mRNA vaccine, successfully treated with prednisolone and rituximab. J Oncol Pharm Pract. 2022;28:1450–3. 35088622 10.1177/10781552221075545

[12] Ai Vuen L Aun Su-Yin E Naila Kori A. Case of acquired Haemophilia A in Southeast Asia following COVID-19 vaccine. BMJ Case Rep. 2022;15:e246922. 10.1136/bcr-2021-246922 PMC8915366 35264381

[13] Farley S Ousley R Van Wagoner N. Autoimmunity after coronavirus disease 2019 (COVID-19) vaccine: a case of acquired hemophilia A. Thromb Haemost. 2021;121:1674–6. 34352911 10.1055/a-1579-5396

[14] Leone MC Canovi S Pilia A. Four cases of acquired hemophilia A following immunization with mRNA BNT162b2 SARS-CoV-2 vaccine. Thromb Res. 2022;211:60–2. 35081484 10.1016/j.thromres.2022.01.017 PMC8770249

[15] Plüß M Mitteldorf C Szuszies CJ. Case report: acquired haemophilia A following mRNA-1273 booster vaccination against SARS-CoV-2 with concurrent diagnosis of pleomorphic dermal sarcoma. Front Immunol. 2022;13:868133. 35479071 10.3389/fimmu.2022.868133 PMC9035784

[16] Lemoine C Giacobbe AG Bonifacino E. A case of acquired haemophilia A in a 70-year-old post COVID-19 vaccine. Haemophilia. 2022;28:e15–7. 34708898 10.1111/hae.14442 PMC8652744

[17] Fu P-A Chen C-W Hsu Y-T. A case of acquired hemophilia A and bullous pemphigoid following SARS-CoV-2 mRNA vaccination. J Formos Med Assoc. 2022;121:1872–6. 35321820 10.1016/j.jfma.2022.02.017 PMC8919791

[18] Cittone MG Battegay R Condoluci A. The statistical risk of diagnosing coincidental acquired hemophilia A following anti-SARS-CoV-2 vaccination. J Thromb Haemost. 2021;19:2360–2. 34101973 10.1111/jth.15421 PMC9771119

[19] Radwi M Farsi S. A case report of acquired hemophilia following COVID-19 vaccine. J Thromb Haemost. 2021;19:1515–8. 33783953 10.1111/jth.15291 PMC8250362

[20] Soliman DS Al Battah A Al Faridi D. Acquired hemophilia A developed post COVID-19 vaccine: an extremely rare complication. J Med Cases. 2022;13:1–4. 35211227 10.14740/jmc3827 PMC8827248

[21] Mahendra A Padiolleau-Lefevre S Kaveri SV. Do proteolytic antibodies complete the panoply of the autoimmune response in acquired haemophilia A? Br J Haematol. 2012;156:3–12. 21988190 10.1111/j.1365-2141.2011.08890.x

[22] Amisha F Saluja P Malik P. Acquired hemophilia A (AHA) due to anti-SARS-CoV-2 vaccination: a systematic review. EJHaem. 2023;4:532–43. 37206259 10.1002/jha2.604 PMC10188482

